# Acid suppressive agents and risk of *Mycobacterium Tuberculosis*: case–control study

**DOI:** 10.1186/1471-230X-14-91

**Published:** 2014-05-13

**Authors:** Wen-Hung Hsu, Chao-Hung Kuo, Sophie SW Wang, Chien-Yu Lu, Chung-Jung Liu, Seng-Kee Chuah, Fu-Chen Kuo, Yen-Hsu Chen, Yaw-Bin Huang, Ming-Feng Hou, Deng-Chyang Wu, Huang-Ming Hu

**Affiliations:** 1 Division of Gastroenterology, Department of Internal Medicine, Kaohsiung Medical University Hospital, Kaohsiung City 807, Taiwan; 2 Department of Medicine, Faculty of Medicine, College of Medicine, Kaohsiung Medical University, Kaohsiung City 807, Taiwan; 3 Division of Gastroenterology, Kaohsiung Chang Gung Memorial Hospital, College of Medicine, Chang Gung University, Kaohsiung City 833, Taiwan; 4 School of Medicine, College of Medicine, E-Da Hospital, I-Shou University, Kaohsiung City 824, Taiwan; 5 Division of Infectious Diseases, Department of Internal Medicine, Kaohsiung Medical University Hospital, Kaohsiung City 807, Taiwan; 6 Graduate Institutes of Clinical Pharmacy, College of Pharmacy, Kaohsiung Medical University, Kaohsiung 807, Taiwan; 7 Department of Surgery, Kaohsiung Medical University Hospital, Kaohsiung City 807, Taiwan; 8 Division of Internal Medicine, Kaohsiung Municipal Hsiao-Kang Hospital, Kaohsiung Medical University, Kaohsiung City 812, Taiwan

**Keywords:** Proton pump inhibitor, Histamine2-receptor antagonist, *Mycobacterium Tuberculosis*, National health insurance database, Taiwan

## Abstract

**Background:**

The acid-suppressive agents have been linked with an increased risk of infectious disease. The relationship between these drugs and *Mycobacterium Tuberculosis* (TB) was not been reported.

**Methods:**

We conducted a case–control study using data from National Health Insurance research database of Taiwan. From 1996 till 2008, and 6541 cases were defined as TB infection/activation (ICD-9 coding plus prescription two of four first-line anti-TB regimen for at least one month). Control subjects who were matched to the TB cases by age and sex were selected with 10:1 ratio. Medical records including acid-suppressive agent prescription and comorbidity, and socioeconomic status were analyzed.

**Results:**

TB infection/activation was more frequent to comorbidity with chronic diseases, alcohol abuse, malignancy, immune deficient/suppression status and acid-related disease (peptic ulcer, reflux esophagitis). Among the TB cases, there was higher exposure record to acid-suppressive agents within 3 months before TB index date (OR 2.43(2.06-2.88) and 1.90 (1.68-2.14) for proton pump inhibitor (PPI) and histamine 2 receptor antagonist (H_2_RA) respectively). After adjusting confounding factors, PPIs prescription 3 months before TB index date had an association of TB infection/activation (adjusted OR 1.63(1.61-1.63)). Similar result was found in H_2_RA user (adjusted OR 1.51(1.50-1.52)). The association of acid-suppressive agents in TB infection/activation was fade gradually when the drug prescription period extended.

**Conclusions:**

Recent prescription of acid-suppressive agent seems to associate the TB infection/activation. In the society where TB was prevalent, evaluation of pulmonary TB before prescription of PPI or H_2_RA is warranted.

## Background

Gastric acid plays an important role in decontamination of upper gastrointestinal tract. Following the widespread use of acid-suppressive agents [1-3], which are the mainstream of treatment in peptic ulcer disease and acid reflux associated esophageal disease, its eliminating acid barrier effect had been implied the association with infectious disease. Proton pump inhibitor (PPI) had marketed for more than two decades, and several studies had mentioned its direct link to several enteric infections, such as *Clostridium diffcile* colitis, *Salmonella* enteritis [4,5] and spontaneous bacterial peritonitis in patients with advanced cirrhosis [6]. In addition, some population- or hospital- based studies of respiratory tract infection also revealed the increased risk of nosocomial and community-acquired pneumonia in patients with PPI use, while the role of histamine 2 receptor antagonist (H_2_RA) was controversial [7-9]. Among variant respiratory tract infectious disease, *Mycobacterium tuberculosis* (TB) infection is still an important health problem in many developing countries. With the characteristics of latent and indolent phases and the emergence of drug resistance [10], the resurgence of TB has great impact on public health [11].

Unlike in developing countries, the disease used to be uncommon in developed countries, but it has re-emerged in the Western countries as a result of the acquired immunodeficiency syndrome (AIDS) epidemic therein as well as the influx of immigrants from developing countries [12,13].

Previous reports have shown a high prevalence of tuberculosis among patients who have undergone gastrectomy, and gastrectomy is considered to be a risk factor for the development of tuberculosis. [14,15] But, the role of gastric acid in TB infection was still unknown.

To our best knowledge, there was no large study which examined the association between acid-suppressive agents and TB. Here, we utilized the database from Taiwan National Health Insurance (NHI) to evaluate its correlation under a population-based, case–control analysis.

## Methods

### Study population and source of data

The NHI program was implemented in Taiwan since 1995, and it offered a comprehensive, unified, and universal health insurance program to all citizens. The state-run Bureau of National Health Insurance (BNHI) contracted with 97% of hospitals as well as 90% of clinics. The BNHI collected all the administrative and claim data between March 1995 and December 2009, these data were anonymous.

The data was analyzed from the National Health Insurance Research Dataset (NHIRD), published by the National Health Research Institute (NHI) in Taiwan.

Taiwan’s National Health Insurance program insures approximately 23 million people, 99% of its population. It offers complete freedom of choice among healthcare providers contracted with the NHI and comprehensive benefits including inpatient care, ambulatory care, dental care, and prescription drugs. The NHI resources, including physician visits, hospital care, and prescribed medications. Using this database, we can identify cohorts, track medical history, establish a prescription drug profile, and set endpoints when researching outcomes. Therefore, the NHIRD is one of the largest and most complete nationwide population-based datasets in Taiwan and there were no statistically significant differences in age, sex, and average insured payroll-related amount between the sample group and all enrollees.

This study used the 1996–2008 National Health Insurance Research Database (NHIRD) that is derived from NHI program which provided a database of 1,000,000 random subjects for research purposes.

Because these were secondary data, each patient’s original identification number has been encrypted to protect privacy by a consistent procedure, so that the linkage of claims belonging to the same patient is feasible within the NHI Research Database.

### Definition of TB infection/activation

We defined active TB from NHRID by compatible ICD-9-CM (International Classification of Disease, 9^th^ Revision, Clinical Modification) codes of TB (010–108) plus the prescription of two of four first-line anti-tuberculosis medications (rifampin, isoniazid, pyrazinamide, ethambutol) for more than 28 days.

All patients who had match TB inclusion criteria were included. Under this definition, both of new symptomatic infection and reactivation who need treatment were included. Theoretically, It also included pulmonary, extra-pulmonary, child and adult. Ultimately, 6,541 patients with TB were included in the study group. Their first ambulatory care visits was assigned as index ambulatory care visits.

### Control group

Matched controls group for this study was likewise extracted from the Registry of Beneficiaries of the NHIRD. We randomly selected 65,410 control subjects (10 for every TB patient), matched with the study group in terms of age, sex and the year and month of index visit.

### Comorbidity

Several underline diseases had been shown to increase the risk of TB infection/activation [16,17], we collected those ICD-9 coding as confounding variables. Furthermore, gastric acid-related gastrointestinal diseases which act as a predictor of using acid-suppressive medication were also included for comorbidity analysis (Table 1). The definition of cormobidity was defined when those ICD-9 coding existed within 3 months before the diagnostic date of TB infection/activation.

**Table 1:** ICD-9-CM codes used for Comorbidity

**Congestive heart failure**	**428**
Coronary heart disease	410-41405
Chronic pulmonary disease	493,494,501-5089
Diabetes Mellitus	2500-2509
End stage renal disease	585
Liver cirrhosis	5712,5715, 5716
Alcohol abuse	291,292,303,304, 305
Peptic ulcer disease	531,532,533,534
Reflux esophagitis	53011,53081
Gastrectomy	534
HIV	042
Connective tissue disease	710, 714, 446, 4476, 1361, 7112,7111,6944,556, 340,3580
Malignant neoplasm	140-208
Organ transplantation	9968

### Exposure of acid-suppressive agents

Acid-suppressive agents included PPI (omeprazole, pantoprazole, lanosoprazole, rabeprazole, esomeprazole) and H_2_RA (cimetidine, ranitidine, famotidine) which were marketed in Taiwan during the observing period. The exposure of acid- suppressive agents was considered when the average dosage was more than half of the defined daily dose (DDD) during the observing period. The defined daily dose (DDD) recommended by the WHO is a unit for measuring a prescribed amount of drug; it is the assumed average maintenance dose per day of a drug consumed for its main indication in adults. We estimated dose–response relationship by using the cumulative amount of PPIs redeemed during the past 90 days, past 180 days, past 1 year and past 2 year as four periods. Cut-off points were greater than 50% DDD and non-use.

### Statistical analysis

Chi-square test was used to compare the distribution of sociodemographic characteristics between patients with and patients without TB. We characterized comorbidity (Table 1) 1 year before index date. The crude and adjusted ORs with 95% confidence intervals (CIs) of exposure for TB cases compared with control subjects were estimated using logistical regression. Previous diagnosis of comorbidity were either risk factors in univariate analyses of tuberculosis or were found to modify the OR for the association between acid suppressive agent and TB by at least 5% if included in a multivariate model. All these priori confounding factor were included in the multivariate analysis. Multivariate analysis was utilized to adjust variable confounding factors in order to identify the association between acid-suppressive agents and TB infection/activation. All the data processing and statistical analysis were performed with SAS 9.3 software.

## Results

Between 1996 and 2008, there were 6541 patients who met the criteria of TB infection/activation. The characteristics of TB cases and controls were presented in Table 2. A p value < 0.05 was considered statistically significant. Similar with previous studies, TB infection/activation was more frequent to comorbid with chronic diseases, alcohol abuse, malignancy and immune deficient/suppression status. Interestingly, TB cases also had higher prevalence of both peptic ulcer disease (16.90% vs. 10.43% of controls, OR 1.75, 95% CI 1.63-1.87) and reflux esophagitis (1.57% vs. 1.02% of controls, OR 1.56, 95% CI 1.27-1.92) within three months before the TB diagnosis. As for the use of acid-suppressive agents, 173 out of 6541 TB cases (2.64%) and 723 out of 65410 controls (1.11%) were exposed to PPI medication within 3 months of diagnosis. During the same period, the use of H_2_RA in TB cases and controls were 5.16% and 2.79%, respectively. After adjusting the confounding variables, their adjusted odds ratio (aOR) of using PPI or H_2_RA in TB cases were1.63 (95% CI 1.61-1.63) (Table 3) and 1.51 (95% CI, 1.50-1.52) (Table 4), respectively.

**Table 2:** Characteristics of cases with TB infection/activation and control subjects

**Characteristics**	**Case n = 6541 (%)**	**Control n = 65410 (%)**	**OR (95%CI)**
Age (mean ± SD)	57.78(±19.55)		
Sex Male/Female	4277/2264	42770/22640	1.0000
Income category			
Monthly income ≦ NT$20000	5371(82.11)	52016(79.52)	0.79-0.90
Monthly income > NT$20000	1170(17.89)	13394(20.48)	
Comorbidity			
Congestive heart failure	304(4.65)	1224(1.87)	2.56(2.25-2.91)
Coronary heart disease	727(11.11)	5674(8.67)	1.32(1.21-1.43)
Chronic pulmonary disease	1087(16.62)	2524(3.86)	4.97(4.60-5.36)
Diabetes mellitus	1274(19.48)	6234(9.53)	2.30(2.14-2.45)
End stage renal disease	182(2.82)	799(1.22)	2.32(1.97-2.73)
Liver cirrhosis	696(10.64)	3952(6.04)	1.85(1.70-2.02)
Alcohol abuse	91(1.39)	229(0.35)	4.02(2.15-5.13)
Peptic ulcer disease	1106(16.90)	6823(10.43)	1.75(1.63-1.87)
Reflux esophagitis	103(1.57)	664(1.02)	1.56(1.27-1.92)
Gastrectomy	23(0.35)	149(0.23)	1.55(1.01-2.40)
HIV	12 (0.18)	3(0.00)	40.04(11.3-141.9)
Connective tissue disease	245(3.75)	1357(2.07)	1.84(1.60-2.11)
Malignant neoplasm	704(10.76)	1906(2.91)	4.02(3.67-4.40)
Organ transplantation	21(0.32)	58(0.08)	3.63(2.20-5.99)
Medication			
PPI	173(2.64)	723(1.11)	2.43(2.06-2.88)
H_2_RA	338(5.16)	1828(2.79)	1.90(1.68-2.14)

*Abbreviations*: TB, *Mycobacterium Tuberculosis*; CI, confidence interval; SD, standard deviation; NT, New Taiwan dollars; OR, odds ratio; HIV, human immunodeficiency virus; PPI, proton pump inhibitor; H_2_RA, histamine2-receptor antagonist.

**Table 3:** Association between variable duration of PPI exposure and TB infection/activation

**Exposure**	**Case (%) n = 6541**	**Control (%) n = 65410**	**Crude OR**	**Adjusted OR**^**†**^
3 months				
Non user	6368(97.36)	64687(98.89)	1	1
User	173(2.64)	723(1.11)	2.43(2.06-2.88)	1.63(1.62-1.63)
6 months				
Non user	6306(96.41)	64276(98.27)	1	1
User	235(3.59)	1134(1.73)	2.11(1.83-2.44)	1.29(1.29-1.30)
12 months				
Non user	6221(95.11)	63575(97.19)	1	1
User	320(4.89)	1835(2.81)	1.78(1.58-2.01)	0.99(0.99-1.01)
24 months				
Non user	6103(93.30)	62519(95.58)	1	1
User	438(6.70)	2891(4.42)	1.55(1.40-1.72)	0.90(0.89-0.90)

*Abbreviations*: OR, odds ratio. †Adjusted for socioeconomic status, congestive heart failure, coronary heart disease, chronic pulmonary disease, diabetes mellitus, end stage renal disease, liver cirrhosis, alcohol abuse, peptic ulcer disease, reflux esophagitis, gastrectomy, HIV, connective tissue disease, malignant neoplasm, organ transplantation.

**Table 4:** **Association between variable duration of H**_**2**_**RA exposure and TB infection/activation**

**Exposure**	**Case (%) n=6541**	**Control (%) n=65410**	**Crude OR**	**Adjusted OR**^**†**^
3 months				
Non user	6203(94.83)	63582(97.21)	1	1
User	338(5.17)	1828(2.79)	1.90(1.68-2.14)	1.51(1.50-1.52)
6 months				
Non user	6073(92.85)	62528(95.59)	1	1
User	468(7.15)	2882(4.41)	1.67(1.51-1.85)	1.29(1.28-1.29)
12 months				
Non user	5847(89.39)	60678(92.77)	1	1
User	694(10.61)	4732(7.23)	1.52(1.40-1.66)	1.15(1.14-1.15)
24 months				
Non user	5549(84.83)	57998(88.67)	1	1
User	992(15.17)	7412(11.33)	1.40(1.30-1.50)	1.06(1.05-1.06)

*Abbreviations*: OR, odds ratio. †Adjusted for socioeconomic status, congestive heart failure, coronary heart disease, chronic pulmonary disease, diabetes mellitus, end stage renal disease, liver cirrhosis, alcohol abuse, peptic ulcer disease, reflux esophagitis, gastrectomy, HIV, connective tissue disease, malignant neoplasm, organ transplantation.

**Figure 1:**
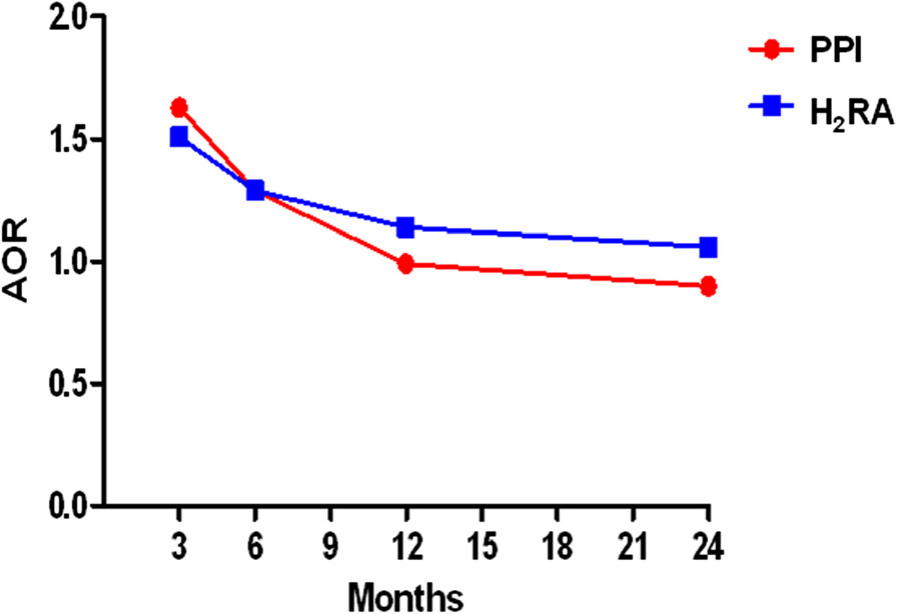
**Association between the using of proton pump inhibitors (PPIs), histamine2-receptor antagonist (H**_**2**_**RA) and *Mycobacterium tuberculosis *infection/activation, according to the timing of acid suppressed agents prescription.** AORs indicate odds ratios.

In order to clarify the association of long-term use of acid-suppressive agents and TB infection/activation, we also observed the prevalence of PPI or H_2_RA use by definition of DDD higher than 50% within 6, 12 and 24 months before TB diagnosis (Tables 3 and 4). The higher possibility of PPI use in TB cases was still noted in 6 months in comparison to controls (aOR, 1.29, 95%CI 1.29-1.30). However, the positive association disappeared in 12 and 24 months observing timeframe (Table 3). Similarly, the decreasing trend of association between H_2_RA and TB following the extension of observing timeframe was also found (Table 4). As the result, the influence of acid-suppressive agents in TB infection/activation was fade gradually when the drug prescription period extended (Figure 1).

## Discussion

Acidic environment of stomach usually keep free from bacteria (except for *Helicobacter pylori*). Somehow, when stomach becomes less acidic, it loses this protective mechanism, and ingested organisms can survive and proliferate.[18]. It also can be the possible reason for the acid-suppressive agent with pneumonia. In Laheij et al. study, which 5,551 cases of community-acquired pneumonia developed in 364,683 people, found that the incidence of pneumonia was about 4.5 times higher in people exposed to acid-suppressive drugs (both PPIs and H_2_RA) than in unexposed individuals.[7]. Subsequent had the similar results discussing the association of PPI with community-acquired pneumonia. [9,19-23]. Patients who developed community acquired pneumonia had higher odds of significant comorbidity conditions, including heart failure and chronic obstructive pulmonary disease. This possible association came to the attention of the general medical community. Although, the risk factors of pneumonia were complicated, the most important risk factor of pneumonia were aging, immunocompromised status and comorbility, [24,25] which may overcome the importance of acid-suppressive agent.

*Mycobacterium tuberculosis* infection can present as an acute process and should be included in the differential diagnosis of community-acquired pneumonia. It is infected by *Mycobacterium tuberculosis* and may mimic classic bacterial pneumonia or masquerade as an atypical pneumonia, with nonproductive cough. Epidemiologic clues *Mycobacterium tuberculosis* share similar risk factor with community acquired pneumonia such as Diabetes mellitus, alcohol consumption, aging and HIV infection. In addition, intravenous drug abuse, immigration from countries of high prevalence, contact with *Mycobacterium tuberculosis* cases were also under higher risk of TB.[11,26,27]. Gastrectomy had also considered an isolated risk factor of *Mycobacterium tuberculosis*.[14,28] Gastrectomy can cause a poor nutritional status leading to poor immunity, and also leads to a poor nutritional status.[29,30]. Long-term PPI therapy has been thought to be associated with micronutrient deficiencies, especially of iron and vitamin B12.[31]. However, the relation between acid suppressive agent and *Mycobacterium tuberculosis* infection was still unknown.

In the present study, the risk of TB infection/activation increased when comorbidity existed, compatible with previous findings. After adjusting these confounding factors, acid-suppressive agents associated with an increasing of TB infection/activation. However, the longer duration the medications were prescribed, the weaker correlation it showed.

Two hypothesis was considered to the statistical association of TB and acid-suppressive agent. One was the acid-suppressive agent had influence to the host immune or nutrition status and cause the host sensitive to TB infection/reactivation. It effect declined with time. The other was initial TB presentation (chronic cough) mimicking acid-related disease and caused the prescription of acid-suppressive agent.

Here we defined acid-suppressive agent exposure as the average dosage during the observing period higher than 50% of DDD. The duration of acid-suppressive agent prescription suggested by NHI guideline was within 4 months after endoscopic diagnosis of peptic ulcers or reflux esophagitis. It seem that acid-suppressive agents were prescribed before TB infection/activation was diagnosed in 3 and 6 months subgroups. Interestingly, in comorbidity analysis, reflux esophagitis was also a risk factor of TB infection/activation. In clinical practice, the acid reflux might be on the list of differential diagnosis for cases who suffered from chronic cough, giving another chance that acid-suppressive agents were taken before anti-TB drug prescription.

The main strength of the present study lies in the use of a true population-based approach, with comprehensive medical records for personal health issue. However, there were some limitations. First, the TB prevalence in the present study was about 49.3 per 100,000 population per year, which was lower than the crude incidence (74.6 per 100,000 population) in Taiwan. [11,32] The reason of underestimated prevalence might come from the definition of TB infection/activation we used, which combined coding system and anti-TB drug prescription record. Inactive TB and cases who had received complete treatment were eliminated from study group. On the contrary, utilizing prescription record could minimize the bias of miscoding. Second, the present study focused on the medical record from NHI research database, which did not include the acid-suppressive agents prescribed from over-the-counter medication. It might underestimate the effect of PPI and H_2_RA. Furthermore, it was also difficult to evaluate the drug compliance. Third, some risk factors associated with immunocompromised status, such as poorly controlled diabetes, underweight, malnutrition, were not available, and these factors were possibly influence the TB activation. Fourth, we separated arbitrarily the using of acid suppressive agent in the interval of 3,6,12,24 months and define the exposure as DDD exceeded 50%. This definition would blur the different influence of long-term, short-term, on-off prescription and on-demand prescription. Further prospective study would warrant to this issue.

Undoubtedly, acid-suppressive agents are of great value for treatment of peptic ulcer disease, gastroesophageal reflux disease, or for prophylaxis against nonsteroid anti-inflammatory drug–related gastrointestinal complications. Following the increase in PPI use in Taiwan [33], the adverse effect of the drug needs to be further noticed. Similar as the possible role in community-acquired pneumonia, recent prescription of acid-suppressive agent seems to associate the TB infection/activation. Under the consideration of initial TB infection/activation mimic atypical reflux esophagitis, in the society where *Mycobacterium tuberculosis* was prevalent, evaluation of pulmonary TB was warranted before prescription of acid-suppressive agent.

## Competing interest

The authors declare that they have no competing interest.

## Authors’ contributions

WHH, CJL: designed the study and analyzed the results and wrote the manuscript. HMH: designed and supervised the study and directed its implementation, including quality assurance and control. CHK, SSWW, FCK, CYL, SKC, YHC, YBH, MFH, DCW: offered the idea of this study and helped in literature review.

## Pre-publication history

The pre-publication history for this paper can be accessed here:

http://www.biomedcentral.com/1471-230X/14/91/prepub
